# Enteric-Coated Cysteamine Bitartrate in Cystinosis Patients

**DOI:** 10.3390/pharmaceutics15071851

**Published:** 2023-06-29

**Authors:** Sabrina Klank, Christina van Stein, Marianne Grüneberg, Chris Ottolenghi, Kerstin K. Rauwolf, Jürgen Grebe, Janine Reunert, Erik Harms, Thorsten Marquardt

**Affiliations:** 1 Department of Paediatrics, Metabolic Diseases, University of Münster, Albert-Schweitzer-Campus 1, 48149 Münster, Germanymarquat@uni-muenster.de (T.M.); 2 UMR 1163, Université Paris Descartes, Sorbonne Paris Cité, Institut IMAGINE, 24 Boulevard du Montparnasse, 75015 Paris, France; 3 Biochimie Métabolomique et Protéomique, Hôpital Necker—Enfants Malades, 149 Rue de Sèvres, 75015 Paris, France; 4 Department of Pediatric Hematology and Oncology, University of Münster, Albert-Schweitzer-Campus 1, 48149 Münster, Germany; 5 Division of Pediatric Oncology, University Children’s Hospital Zürich, Steinwiesstraße 75, 8032 Zürich, Switzerland

**Keywords:** cystinosis, enteric-coated cysteamine, immediate-release cysteamine (Cystagon^®^), cystine-levels, pharmacokinetics

## Abstract

Cystinosis is a severe inherited metabolic storage disease caused by the lysosomal accumulation of cystine. Lifelong therapy with the drug cysteamine bitartrate is necessary. Cysteamine cleaves intralysosomal cystine, and thereafter, it can exit from the organelle. The need for frequent dosing every 6 h and the high prevalence of gastrointestinal side effects lead to poor therapy adherence. The purpose of our study was to improve cysteamine treatment by comparing the efficacy of two cysteamine formulas. This is highly relevant for the long-term outcome of cystinosis patients. The cystine and cysteamine levels of 17 patients taking immediate-release cysteamine (IR-cysteamine/Cystagon^®^) and 6 patients taking encapsulated delayed-release cysteamine (EC-cysteamine) were analyzed. The EC-cysteamine levels showed a near-ideal pharmacokinetic profile indicative of delayed release (longer T_max_ and T_min_), and the corresponding cystine levels showed few fluctuations. In addition, the C_max_ of IR-cysteamine was greater, which was responsible for unbearable side effects (e.g., nausea, vomiting, halitosis, lethargy). Treatment with EC-cysteamine improves the quality of life of cystinosis patients because the frequency of intake can be reduced to 2–3 times daily and it has a more favorable pharmacokinetic profile than IR-cysteamine. In particular, cystinosis patients with no access to the only approved delayed-release cysteamine Procysbi^®^ could benefit from a cost-effective alternative.

## 1. Introduction

Cystinosis is an ultrarare, autosomal-recessive lysosomal storage disease caused by mutations in the CTNS gene, which encodes a lysosomal transmembrane protein called cystinosin [1,2,3,4]. The impairment of transporter function leads to the accumulation of the poorly soluble amino acid cystine in various tissues, causing cell and tissue damage [1,5]. Without any treatment, the majority of cystinosis patients can either die early from dehydration or electrolyte disturbances or develop end-stage renal failure until the age of ten years, requiring dialysis or kidney transplantation [2]. Current therapy relies on symptomatic treatment including dialysis, renal transplantation, and cysteamine application. A curative therapy is not available.

Cysteamine bitartrate depletes cystine in lysosomes by reacting with cystine and forming a mixed cysteine–cysteamine disulfide that is able to exit the lysosomes via lysine transporters [3,4,5]. Via the analysis of white blood cell cystine concentrations, the body cystine concentrations can be estimated, which are used to monitor the efficacy of cysteamine therapy. The long-term outcome of cystinosis patients can be improved by maintaining low intracellular cystine concentrations, which is achieved by the regular intake of cysteamine bitartrate every 6 h [6]. This strict treatment regime, especially the required intake during the night [7,8] together with various adverse side effects like body odor and halitosis, nausea, vomiting, and diarrhea, leads to poor therapy adherence and compliance problems [7,9,10,11]. 

Currently, three different formulations of oral cysteamine bitartrate are available: immediate-release (IR-) cysteamine bitartrate (Cystagon^®^, Mylan Pharmaceuticals, Canonsburg, PA and Recordati Pharma GmbH) [7,8,12,13], delayed-release (DR-) enteric-coated cysteamine bitartrate, RP103 (Procysbi^®^, Horizon Pharma USA and Chiesi Farmaceutici S.p.A., Parma, Italy) [14,15,16,17], and a third formulation in which cysteamine is encapsulated by a pharmacist for enteric release (EC-cysteamine). In Germany, this formulation was generated from the patient advocacy group due to unbearable side effects of IR-cysteamine (Cystagon^®^). It was started as an alternative to Cystagon^®^ before Procysbi^®^ appeared on the market. Cysteamine bitartrate is filled into enteric-release capsules and sealed with a fluid. Having been used in clinical practice for many years, a delayed release effect has been described [18] with fewer side effects in comparison to Cystagon^®^ [19,20,21,22]. 

The simultaneous ingestion of cysteamine bitartrate formulations with food leads to large fluctuations in plasma cysteamine levels, and absorption may be reduced by 30% [23], especially compared to food intake 2 h after administration [17]. There are no differences when cysteamine is taken with water or applesauce/orange juice; on the other hand, particularly fatty foods impede cysteamine absorption [24]. The highest cysteamine levels are reached after absorption in the small intestine/duodenum, while less is absorbed in the stomach and even less in the cecum [25]. From a purely pharmacokinetic point of view, this argues for the development of delayed release formulations in the small intestine such as Procysbi^®^ and EC-cysteamine. According to the pharmacokinetic information in the package inserts of Cystagon^®^ (IR) and Procysbi^®^ (DR), cysteamine bitartrate is moderately bound to human plasma proteins, mainly albumin, with a mean protein binding of 52%. Plasma protein binding is within the range of concentrations achieved clinically with recommended doses, regardless of concentration [12,17]. The volume of distribution (Vd/F) is 382 l for DR and higher compared to 198 l for IR cysteamine bitartrate [17]. The apparent clearance (Cl/F) of cysteamine is similar between DR (1.2 ± 0.8 L/min) and IR cysteamine bitartrate (1.4 ± 0.8 L/min). The half-life of 253 min for DR and 90 min for IR cysteamine bitartrate reflects the delayed release [17]. The urinary excretion of unchanged cysteamine is less than 1.7% of the total daily dose, while most cysteamine is excreted as sulphate [12].

Improving the strict therapeutic schedule and reducing the severe side effects, especially when IR-cysteamine is administered, are necessary to improve treatment compliance and long-term outcome in cystinosis patients, and is the purpose of our study. We report the pharmacokinetics of cysteamine bitartrate enterically coated by a pharmacist in comparison with IR-cysteamine bitartrate in a cohort of cystinosis patients. In addition, we analyze treatment compliance and adverse side effects. 

## 2. Materials and Methods

Patient data were analyzed retrospectively from health records that had been collected over the period of three years as part of routine care: cystinosis patients in our department receive a one-time individual determination of their cysteamine and cystine kinetics (standard operating procedure) in order to optimize the therapy such as individual dosing schedules, medication doses, and intervals of medication intake as well as the reduction in side effects.

### 2.1. Subjects

The data of 17 patients taking IR-cysteamine bitartrate (Cystagon^®^) and 6 patients taking EC-cysteamine bitartrate were used. For three patients, the data for both preparations were available. Table 1 provides an overview of the more detailed patient characteristics. All 23 patients had received regular long-term therapy with cysteamine since their early childhood when cystinosis was diagnosed. The six patients under EC-cysteamine treatment had been taking this formulation for the last 5–10 years.

### 2.2. Data Collection and Dosages

Blood samples were collected from a temporary peripheral venous line. Measurements were taken for 6 h when taking IR-cysteamine and up to 12 h when taking EC-cysteamine (Table 2). This difference in 6 h was defined because of the estimated delayed-release effect of EC-cysteamine. The patients took their respective (single) morning dose of IR-cysteamine (Cystagon^®^) or EC-cysteamine on an empty stomach with water or applesauce. Food was permitted two hours before and after the first blood collection. After 2 h, the patients were allowed to eat something they were used to in their daily lives (e.g., a light breakfast, lunch, dinner, or snacks). All concomitant medications were taken regularly during the blood collection time. The last regular dose of the respective cysteamine formula was the evening before the measurement day to avoid interferences. The patients were asked to indicate potential side effects during the measurement phases. 

Individual dosages were adjusted to keep intracellular cystine levels in the recommended range, in accordance with the prescribing information. In general, up to 12 years of age, body surface area is used for dosage calculation (standard dose: 1.30 g/m²). Older patients or patients weighing over 50 kg receive 2 g per day up to a maximum of 1.95 g/m²/day. IR-cysteamine and EC-cysteamine were taken with a frequency of four times in 24 h.

### 2.3. Enteric-Coated Cysteamine Bitartrate (EC-Cysteamine)

Capsules of commercial cysteamine bitartrate usually dissolve in the stomach where they release cysteamine bitartrate, causing adverse side effects. In order to delay this process and achieve intestinal release, cysteamine bitartrate was filled into enteric-release capsules that were additionally sealed with a fluid containing Eudragit S12.5 and pharmaceutical acetone. All six patients received their EC-cysteamine bitartrate as a prescription drug from their local pharmacy, which performed the coating process. This is a common practice in Germany. The pharmacies used coating kits from two different pharmaceutical manufacturers: appropharm^®^ (Paris, France) and Iphas^®^ Pharma-Verpackung GmbH (Würselen, Germany). The gastric-resistant capsules of both kits (Size: 00) were manually coated once with the sealing fluid.

### 2.4. Cystine Measurement

Heparinized plasma was analyzed for cysteamine concentration and EDTA-blood-derived leucocytes were used to determine the intracellular cystine concentrations. 

The leucocytes were isolated by sedimentation. Cell pellets were resuspended in a phosphate buffer containing N-ethylmaleimide (NEM) to alkylate-free sulfhydryl groups. Deproteinization was conducted by acidification with 12% sulfosalicylic acid. Norvaline was added as an internal standard and protein concentrations measured via Lowry’s assay [26]. The amino acid mixture was analyzed by column chromatography as described by Spackman et al. [27] with photometric concentration determinations of cystine and norvaline.

Cystine values can also be specified in the unit nmol hemicystine/mg protein, whereas our laboratory standard is in nmol cystine/mg protein and was used in this work. The therapeutic goal under 0.5 nmol cystine/mg protein (=1 nmol hemicystine/mg protein) for cystine levels in white blood cells was established previously [10].

### 2.5. Cysteamine Measurement

Cysteamine measurements were carried out at the Metabolic Biochemistry Laboratory, University of Paris-Descartes (Hospital Necker Enfants Malades). Cysteamine plasma concentrations were determined as described previously via liquid chromatography MS/MS [28] and are expressed in µmol/L.

### 2.6. Statistical Analysis

Mean levels, standard deviation ranges, and *p*-values were calculated using GraphPad Prism Version 9.1.0. C_max_, T_max_ and areas under the curve were calculated with the Microsoft Excel 2016 version for Windows. The Wilcoxon test was used to detect statistical significance (*p*-value lower than 0.05).

### 2.7. Limitations

One of the six patients under EC-cysteamine therapy had high initial cystine values due to a misunderstanding of the dosing schedule before the blood collection day. We excluded these values from the mean levels.

### 2.8. Consent of Local Ethic Board

Data analysis was consented by the local ethics board (number 2019-199-f-S) and informed consent was obtained.

## 3. Results

### 3.1. Patient Characteristics

This retrospective analysis includes 23 cystinosis patients. Seventeen patients received IR-cysteamine and six received EC-cysteamine. The mean age of the patients receiving IR-cysteamine was 22.35 years [range: 11–49] and 21.5 years [range: 14–29] in the group of EC-cysteamine patients. The mean single cysteamine dose in the IR-cysteamine cohort was 586.03 mg [range: 300–1050] and in the EC-cysteamine cohort was 625 mg [range: 450–900]. Further patient characteristics are summarized in Table 1. Overall, there was no significant difference in the patient characteristics between the two cohorts.

### 3.2. Mean Plasma Cysteamine Levels

**Figure 1:**
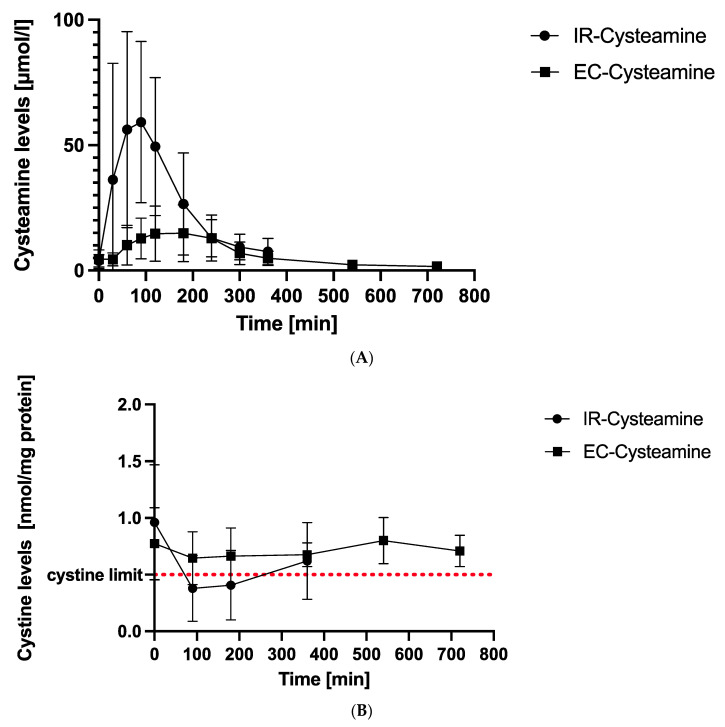
(**A**) Mean cysteamine levels. This figure demonstrates mean cysteamine levels at different time points after intake of IR-cysteamine (dots) and EC-cysteamine (boxes) in µ/mol/L with standard deviations (SD). Peak values are reached after 90 min for IR-cysteamine and 180 min for EC-cysteamine after a latency period of 30 min. (**B**) Mean cystine levels. This figure illustrates mean cystine values in white blood cells (WBC) with SD at different time points. IR-cysteamine (dots) showed a minimum value after 90 min and was the only formulation that reached the recommended upper cystine concentration with therapy of 0.5 nmol/mg protein (red line). EC-cysteamine (boxes) reflected a steady-state characteristic without falling below the target limit of 0.5 nmol/mg protein.

We compared the mean cysteamine levels after a single dose of IR-cysteamine and EC-cysteamine, which are presented in Figure 1A. The corresponding data are shown in Table 3.

The plasma cysteamine concentrations after IR-cysteamine (Cystagon^®^) intake rose from 3.89 ± 2.39 µmol/L up to a peak value of 59.25 ± 32.17 µmol/L after 90 min and decreased to its final value at 360 min (7.53 ± 5.23 µmol/L). 

EC-cysteamine showed a latency period of 30 min (zero value: 4.54 ± 3.65 µmol/L, 30 min: 4.50 ± 2.58 µmol/L) and began to rise to a peak value at 180 min (14.9 ± 11.35 µmol/L). It decreased to its final value at 720 min (1.65 ± 0.7 µmol/L).

### 3.3. Mean Cystine Levels in WBC

The mean cystine levels under IR-cysteamine and EC-cysteamine were compared at corresponding measurement times and are graphically illustrated in Figure 1B. The exact values including standard deviations (SDs) are presented in Table 4.

After IR-cysteamine intake, intracellular cystine levels decreased from 0.96 ± 0.51 nmol/mg protein to a minimum of 0.38 ± 0.29 nmol/mg protein after 90 min. After 90 min, the cystine had already increased again to a maximum value after 360 min of 0.62 ± 0.34 nmol/mg protein. 

In contrast, the mean curve of EC-cysteamine showed only small fluctuations over 12 h. There was a slight decrease initially from zero (0.77 ± 0.32 nmol/mg protein) to 90 min (0.65 ± 0.23 nmol/mg protein) and a slight increase from 380 min (0.67 ± 0.10 nmol/mg protein) to 540 min (0.8 ± 0.20 nmol/mg protein). The last value at 720 min (0.71 ± 0.14 nmol/mg protein) was lower again and in the same range as the 90–180–360 min values. A significant turning point or minimum is not visible. The curve seems to hover around the 0.7 nmol/mg protein value and has a total fluctuation range of 0.15 nmol/mg protein.

Only IR-cysteamine reached the recommended upper target value of 0.5 nmol/mg protein.

### 3.4. Individual Curves of Cystine Levels in EC-Cysteamine Patients

**Figure 2:**
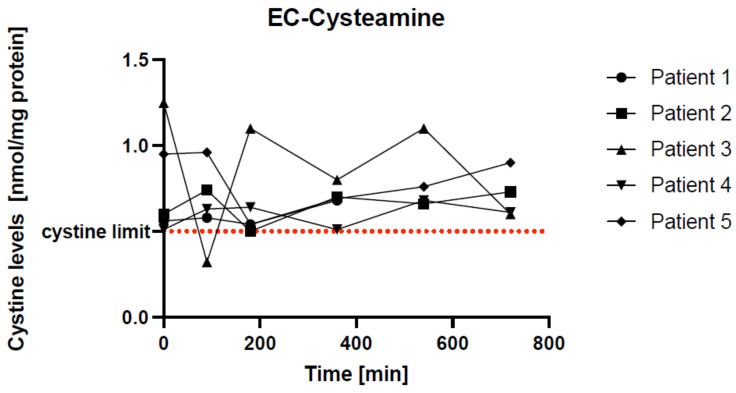
Individual curves of cystine levels in five EC-cysteamine patients. The red line represents the cystine limit of 0.5 nmol/mg protein. Every single patient reached this target limit at different times: patients 1, 2, and 5 after 180 min, patient 3 after 90 min, and patient 4 after 360 min. Patients 1, 2, and 4 had an ideal pharmacokinetic profile with small fluctuations.

As shown in Figure 2, there were some differences in the individual cystine curves. 

Patients 1, 2, and 4 showed similar curves overall. Patients 1 and 2 reached a minimum cystine concentration after 180 min and were within the therapeutic target value of 0.5 nmol/mg protein at that time. Patient 4 reached the therapeutic target value at baseline and after 360 min. All had minimal fluctuations around the cystine threshold. This indicates good drug adaptation.

Patients 3 and 5 started with higher baseline values. Patient 3 reached the therapeutic target after 90 min. From then on, the values fluctuated widely, with the last value being lower again after 720 min. Patient 5’s values fell below the cystine threshold after 90 min and then rose again.

### 3.5. Pharmacokinetic Analysis

As pictured in Table 5, the mean maximum serum concentration of cysteamine (C_max_) after the ingestion of EC-cysteamine (20.4 ± 7.64 µmol/L) was significantly lower than that of IR-cysteamine (74.55 ± 45.7 µmol/L) (*p*: 0.002), while the mean time to reach C_max_ (T_max_) was shorter with IR-cysteamine (75.88 ± 23.99 min) than with EC-cysteamine (120 ± 70.36 min). The mean T_max_ values were not statistically different (*p*: 0.104). 

The mean area under the curve (AUC) of cysteamine was calculated both after one dosing (6 h IR: 9991 and 12 h EC: 4792) and for 24 h (IR: 39,964 vs. EC: 9584) for better comparability. The AUC for IR-cysteamine was four times greater than for EC-cysteamine within 24 h.

The mean minimum cystine concentrations in WBC (C_trough_) were below 0.5 nmol cysteine/mg protein for both drugs in the therapeutic range (EC: 0.48 ± 0.09 nmol/mg protein vs. IR: 0.35 ± 0.20 nmol/mg protein). The mean time (T_min_) to reach C_trough_ was significantly different (*p*: 0.032) and was almost 80 min longer following EC-cysteamine (EC: 198 ± 98.59 min vs. IR: 116 ± 42.27 min).

### 3.6. Side Effects

In terms of side effects, 15 of 17 patients (88.2%) with IR-cysteamine (Cystagon^®^) reported gastrointestinal side effects like nausea, vomiting, abdominal pain, and sulfurous body odor or halitosis. 

With EC-cysteamine, not a single patient (0/6) suffered from nausea and vomiting. Four patients (66.7%) still noticed sulfurous body odor or halitosis, but all of them specified this adverse side effect as being less uncomfortable.

## 4. Discussion

In this study, we compared EC-cysteamine, an enteric-release formulation provided as a prescription drug by German pharmacies, with conventional IR-cysteamine (Cystagon^®^). Only long-term cystine concentrations below the target goal of 0.5 nmol cystine/mg protein can stop the progression of cystinosis. Therefore, cystinosis patients require a strict, lifelong cysteamine therapy regimen as the best option to prevent or at least delay end-stage renal failure and other cystinosis complications. This strict regime involves IR-cysteamine (Cystagon^®^) intake every 6 h, even during the night. Young children must swallow numerous large capsules [7,8]. According to the package leaflet of IR cysteamine (Cystagon^®^), severe gastrointestinal side effects such as nausea, vomiting, and diarrhea are very common. The same applies to lethargy and anorexia, which is likely to be particularly challenging in children and adolescents in addition to the burden of the disease itself. Abdominal pain or headache are common, as are bad breath and body odor. Occasionally, irreversible skin and bone changes also occur, as well as central nervous phenomena such as hallucinations and nervousness [12]. All of this challenges patients’ and parents’ everyday life enormously, which leads to poor adherence and compliance, and finally results in the premature progression of cystinosis [29,30]. Levtchenko et al. demonstrated that only 23% (5 of 22) of all patients adhered to the strict dosing interval of 6 h under IR-cysteamine treatment [8]. Therefore, a cysteamine formulation with an improved pharmacokinetic profile and tolerability is highly warranted. 

Today, it is well known that cysteamine release from the small intestine instead of the stomach has preferable pharmacokinetics with a greater C_max_ and cystine decrease in WBC, which is achieved by a retarding effect [14,18,25,31]. 

Our data prove the expected intestinal delayed release of EC-cysteamine in comparison to IR-cysteamine, which is reflected by the longer mean T_max_ of around 45 min (EC: 120 min vs. IR: 76 min). This is consistent with two previous studies using similar enteric-coated cysteamine bitartrate [18,19], as well as for IR-cysteamine [22,32]. The delayed release effect of EC-cysteamine was further indicated by the longer mean T_min_ of approximately 80 min, which was statistically significant (*p*: 0.032) (cf. Table 5). The mean minimum concentrations of cystine in WBCs (mean C_trough_) showed no statistically significant differences and were both within the therapeutic range, proving an adequate decrease with both formulations. 

The mean maximum cystine decrease in white blood cells (WBCs) was 60% with IR-cysteamine, which is in line with previous studies [33], and ‘only’ 16.5% with EC-cysteamine. The maximum cystine decrement in WBCs seems to depend on single dosing vs. steady state dosing: Belldina determined a lower maximum decrease of 48% following IR-cysteamine in a steady-state condition [32], whereas we measured a decrease of 66.7% following EC-cysteamine (single-dose characteristic) in our outlier patient. We excluded this patient with too-high cystine 0 values from the mean because of a miscommunication in the fasting period, which was longer than 12 h (0 min: 3.9 nmol cysteine/mg protein, 540 min: 1.3 nmol cysteine/mg protein = C_trough_). Of note, the outlier cysteamine values were in the mean (0 min: 5.9 µmol/L, 180 min: 18.2 µmol/L (C_max_), 540 min: 5.4 µmol/L). This single course indicates that despite the same C_max_ and AUC of the drug with this single-dose characteristic, the ability to lower cystine levels in WBCs is increased. Its cystine levels do not reach the therapeutic range, which would seem to require a higher starting dose or a shorter dosing interval.

An important control parameter in the long-term measurements is the time in which cystine levels can be kept in the therapeutic range under 0.5 mg cysteine/mg protein. The mean cystine values following IR-cysteamine intake were, overall, 187 min in the adequate range and rose again after 258 min. Contrary to the measurement of Belldina [32] and the recommendation in the package leaflet of Cystagon^®^ [13], we could not prove that the target ranges are maintained over 8 h. This reveals an underdosage in our patients. The mean cystine values with EC-cysteamine were not in the therapeutic range at all. Unfortunately, the mean values are strongly influenced by the individual values of the small patient cohort. As already described, the mean C_trough_ of EC-cysteamine in WBC was well within the therapeutic range. Nevertheless, the individual cystine curves also showed underdosing here. Of note was the time course: the 0-value and the last value after 720 min are comparably high, without a turning point being recognizable until then. This indicates a steady-state characteristic. 

As illustrated in Table 5, the observed mean maximum cysteamine concentrations (C_max_) differed significantly in our study. The C_max_ was more than three times higher with IR- than with EC-cysteamine. The AUC of IR was approximately twofold higher after one dosage. This indicates a lower bioavailability of EC-cysteamine. Gangoiti et al. examined pharmacokinetic parameters following 450 mg and 900 mg enteric release cysteamine doses (450 mg EC: C_max_ 41.6 µmol/L, T_max_ 220 min, AUC 5194 vs. 900 mg EC: C_max_ 84.2 µmol/L, T_max_ 255 min, AUC 11,814) [18]. It shows that the bioavailability (AUC) can be increased in a dose-dependent manner. Thus, a higher C_max_ and even a prolonged T_max_ can be achieved. The same effects could be proven by Dohil comparing a single with a double dose of enteric-release cysteamine [19]. 

The pharmacokinetic parameters reported by Dohil et al. were comparable after the intake of their single-dose enteric-release cysteamine (n = 7): C_max_ 46.4 µmol/L, T_max_ 176 min and an AUC of 5194. The dosages were equivalent to our patients at 45 mg/kg body weight per day [19]. In this case, our AUC is almost identical (EC: 4972), which confirms a comparable bioavailability. However, our C_max_ is only half as large with a shortened T_max_. An explanation for this is offered by the individual curves of the seven participants, which showed a shorter plateau phase of cysteamine levels. The individual courses of the cystine contents in WBC were similar and partly subject to unpredictable fluctuations. We support the authors’ considerations that there is a correlation with individual intestinal dysmotility like constipation or delayed gastric release as well as a batch variability of capsules. One of our patients had an intestinal perforation after five years of regular EC-cysteamine bitartrate ingestion. During the emergency operation, surgeons detected undissolved EC-capsules and capsule remnants in the perforation area. EC-cysteamine bitartrate was suspected as having a similar slowing-down effect on bowel motility as cysteamine hydrochloride (HCl) [19]. Cysteamine-HCl revealed an inhibiting effect on the intestines in a rat model [34] and, furthermore, led to histological changes up to duodenal ulcer in rat mucosa, which could result in a perforation [35].

Moreover, Dohil et al. demonstrated that the twice-daily dosing of enteric-release cysteamine plus a dose reduction up to 62% of the corresponding IR-cysteamine dose effectively kept WBC cystine levels under the threshold in a steady-state design [19]. However, since our patients with EC-cysteamine prescriptions had been taking this preparation for several years, one could assume a steady state. Furthermore, our patients did not engage in dose reduction and took it more frequently than twice daily. The reasons why our EC-cysteamine-adjusted patients were nevertheless not in the therapeutic range were a slight underdosing and uncertain medication adherence in the past. A twice-daily dose seems to be ineffective in the mean because of too-high 0-values after the fasting period. However, with respect to the individual curves of the cystine levels, patients 1, 2, and 4 were in the therapeutic range and a steady-state condition was achieved over 12 h. We state that well-controlled EC-cysteamine patients with good adherence to therapy achieve pharmacokinetics at steady-state, which allows a lower dose interval of 2x daily. Levels need to be further monitored to avoid underdosing. If one or more doses are skipped or missed, either a higher dose of EC-cysteamine has to be taken to compensate, or it has to be taken initially again every 6–8 h.

In summary, we cannot recommend reducing the dose for EC-cysteamine to 62% in comparison to IR-cysteamine in our patient group. A dose adjustment down to three instead of four times per day was possible. Individual measurements of cysteamine and cystine blood levels must be obtained regularly, even in drug adjustment phases. With good adherence to EC-cysteamine, a further dose interval reduction down to twice per day might be possible. 

There is only one publication on the long-term outcome of EC-cysteamine in cystinosis patients. A 6-year follow-up showed that twice-daily administration resulted in no change in the mean WBC cystine levels or deterioration in the estimated glomerular filtration rate [21]. As in our study, the patient cohort (n = 2) was small. Shortly thereafter, Procysbi^®^ with its delayed-release effect appeared on the market and EC-cysteamine was not pursued any further.

We confirm the theory that the C_max_ correlates with the occurrence of side events [18,25]. EC-cysteamine was better tolerated (IR: 88.2% nausea, vomiting, halitosis, and sulfurous body odor vs. EC: 0% nausea or vomiting, but 66.7% still with mild halitosis and body odor). Please note that our study was not blinded, which could have influenced the patient findings. Finally, we speculate that an optimally tolerated cysteamine preparation must have a retarding effect with a particularly long-lasting plateau phase in release, at an overall lower concentration (C_max_), so that the AUC and bioavailability remain identical to immediate release preparations. But this drug does not (yet) exist. Further studies are needed.

There are considerations for the future as to how the pharmacokinetics and tolerability of cysteamine can be further improved. An encapsulation of the granules, as in Procysbi^®^, combined with a non-pH-dependent capsule (PO-001) leading to a sustained-release effect was tested by Berends et al. in 2020 [36]. The cysteamine formulation PO-001 showed a decrease in peak-through variability with fewer adverse events in healthy male adults in comparison to Procysbi^®^ and Cystagon^®^ as well as a further extended-release effect than Procysbi^®^. Twice-daily administration of PO-001 was possible, but the results showed a lower bioavailability compared to Procysbi^®^ and Cystagon^®^. The same effect of a reduced AUC and bioavailability was obvious in our study with EC-cysteamine. We can confirm Berend et al.’s. considerations that the enteric coating (both with Eudragit^®^, Germany) was the cause of this and that a more adapted coating could reduce the delay in the release to enhance the bioavailability [36]. Other variants of Eudragit^®^ for coating exist and could be studied to achieve an even better cysteamine release in the intestinal tract.

Currently, the only FDA-approved twice-daily cysteamine formulation is Procysbi^®^ [17]. Various studies have already shown superiority to Cystagon^®^ in terms of tolerability, effectiveness, and pharmacokinetics [14,16,22,37]. Switching from IR- to DR-cysteamine is also recommended [38]. However, there are isolated cases in which Procysbi^®^ caused more adverse events [14] or led to a deterioration in kidney function; in 2017, Bäumner et al. reported two patients with decreased disease control and an increase in adverse side effects after switching from IR- to DR-cysteamine [39]. For these patients, the only option left was tolerating pronounced adverse events twice daily or the application of IR-cysteamine with nocturnal interruptions. Delivery bottlenecks for medicines, inflation, and a lack of raw materials will probably shape life in the 21st century more than before. Especially in countries without access to Procysbi^®^ or as a financially more favorable formulation in context of global scarcity of resources, EC-cysteamine might be relevant. To clarify, our study does not intend nor is empowered to compare EC-cysteamine to Procysbi^®^, but it should not be forgotten as another alternative formulation.

Cystinosis is an ultrarare disease. In Germany, there are approximately 150 patients with diagnosed nephropathic cystinosis. The subgroup of EC-cysteamine prescription is even smaller. Therefore, we were not able to achieve nine participants, which is necessary to give pharmacokinetic properties for a drug [34]. But despite all of this, the findings emphasize that the individual monitoring of pharmacokinetics is necessary and the only way to gain therapy control.

## 5. Conclusions

To conclude, we recommend therapy for cystinosis patients with a delayed-release formulation like Procysbi^®^ [22]. If this is not well tolerated, EC-cysteamine could be used as a safe alternative to prevent non-adherence to therapy from deteriorating mortality and long-term outcome. In particular, cystinosis patients with no access to Procysbi^®^ could benefit from a cost-effective formulation that is well tolerated and pharmacokinetically efficient. EC-cysteamine treatment improves the quality of life of cystinosis patients as it allows a dosing frequency of 2–3 times per day and has a preferable tolerability and pharmacokinetic profile compared to IR-cysteamine (Cystagon^®^).

## Figures and Tables

**Table 1:** Patient characteristics. ns: not statistically significant.

Spalte1	IR-Cysteamine (Cystagon^®^)	EC-Cysteamine	*p*-Value
Number of patients	17	6	
Mean Age (years)	22.35 [range: 11–49]	21.5 [range: 14–29]	ns
Weight (kg)	54.53 [range: 28–112]	57.67 [range: 40–74]	ns
Female/Male	5/12	2/4	
Mean single cysteamine dose (mg)	586.03 [range: 300–1050]	625 [range: 450–900]	ns
Number of patients with kidney transplantation	8	4	

**Table 2:** Blood collection schedule.

Time (Minutes)	10 mL EDTA Blood	1 mL Heparinized Plasma
0 (before cysteamine intake)	X	X
30		X
60		X
90	X	X
120		X
180	X	X
240		X
300		X
360	X	X
540 *	X *	X *
720 *	X *	X *

* Only EC-cysteamine.

**Table 3:** Mean cysteamine levels. This table shows mean cysteamine levels in µ/mol/L including SD at the corresponding measurement period of 360 min for IR-cysteamine and of 720 min for EC-cysteamine after a single dose. (* *p*-value < 0.05, ns: not statistically significant).

Time (Minutes)	IR-Cysteamine (Cystagon^®^)	EC-Cysteamine	*p*-Values
0	3.89 ± 2.39	4.54 ± 3.65	ns (0.99)
30	36.18 ± 46.43	4.50 ± 2.58	* (0.0022)
60	56.21 ± 39.06	10.16 ± 7.99	* (0.0032)
90	59.25 ± 32.17	12.8 ± 8.1	* (0.0014)
120	49.43 ± 27.54	14.72 ± 11.04	* (0.029)
180	26.57 ± 20.34	14.9 ± 11.35	ns (0.19)
240	12.96 ± 9.20	12.9 ± 7.42	ns (0.87)
300	9.34 ± 5.10	6.88 ± 4.47	ns (0.27)
360	7.53 ± 5.23	4.91 ± 2.87	ns (0.22)
540		2.33 ± 1.05	
720		1.65 ± 0.70	

**Table 4:** Mean cystine levels. This table shows the mean cystine levels (nmol cystine/mg protein) after the intake of a single dose of IR-cysteamine (Cystagon^®^) and EC-cysteamine including SD at corresponding measurement times. (* *p*-value < 0.05, ns: not statistically significant).

Time (Minutes)	IR-Cysteamine (Cystagon^®^)	EC-Cysteamine	*p*-Values
0	0.96 ± 0.51	0.77 ± 0.32	ns (0.059)
90	0.38 ± 0.29	0.65 ± 0.23	ns (0.059)
180	0.41 ± 0.31	0.66 ± 0.25	* (0.03)
360	0.62 ± 0.34	0.68 ± 0.10	ns (0.63)
540		0.8 ± 0.21	
720		0.71 ± 0.14	

**Table 5:** Pharmacokinetic parameters. This table demonstrates the pharmacokinetic parameters in the cystinosis patients after the oral administration of a single dose of IR- and EC-cysteamine. C_max_: maximum concentration of cysteamine, T_max_: time until C_max_ is reached, AUC: area under the curve, C_trough_: minimum concentration of cystine in WBC, T_min_: time until C_trough_ is reached, * *p*-value < 0.05, ns: not statistically significant.

Spalte1	IR-Cysteamine (Cystagon^®^)	EC-Cysteamine	*p*-Value
Mean C_max_ (µmol/L)	74.55 ± 45.70 (range 4–122)	20.4 ± 7.64 (range: 13.1–32.1)	* (0.0022)
Mean T_max_ (minutes)	75.88 ± 23.99 (range: 30–120)	120 ± 70.36 (range: 60–240)	ns (0.104)
Mean time with cystine values under 0.5 nmol/mg protein (minutes)	186.72	0	
AUC cysteamine (after 1 dose)	9991	4792	
AUC cysteamine (24 h)	39,964	9584	
Decrease of cystine content in WBC (%)	60.42	16.5	
Mean C_trough_ (nmol/mg protein)	0.35 ± 0.29 (range: 0.05–0.98)	0.48 ± 0.09 (range: 0.32–0.54)	ns (0.0672)
Mean T_min_ (minutes)	116 ± 42.27 (range: 90–180)	198 ± 98.59 (range: 90–360)	* (0.0324)

## Data Availability

The data presented in this study are available on request from the corresponding authors.
